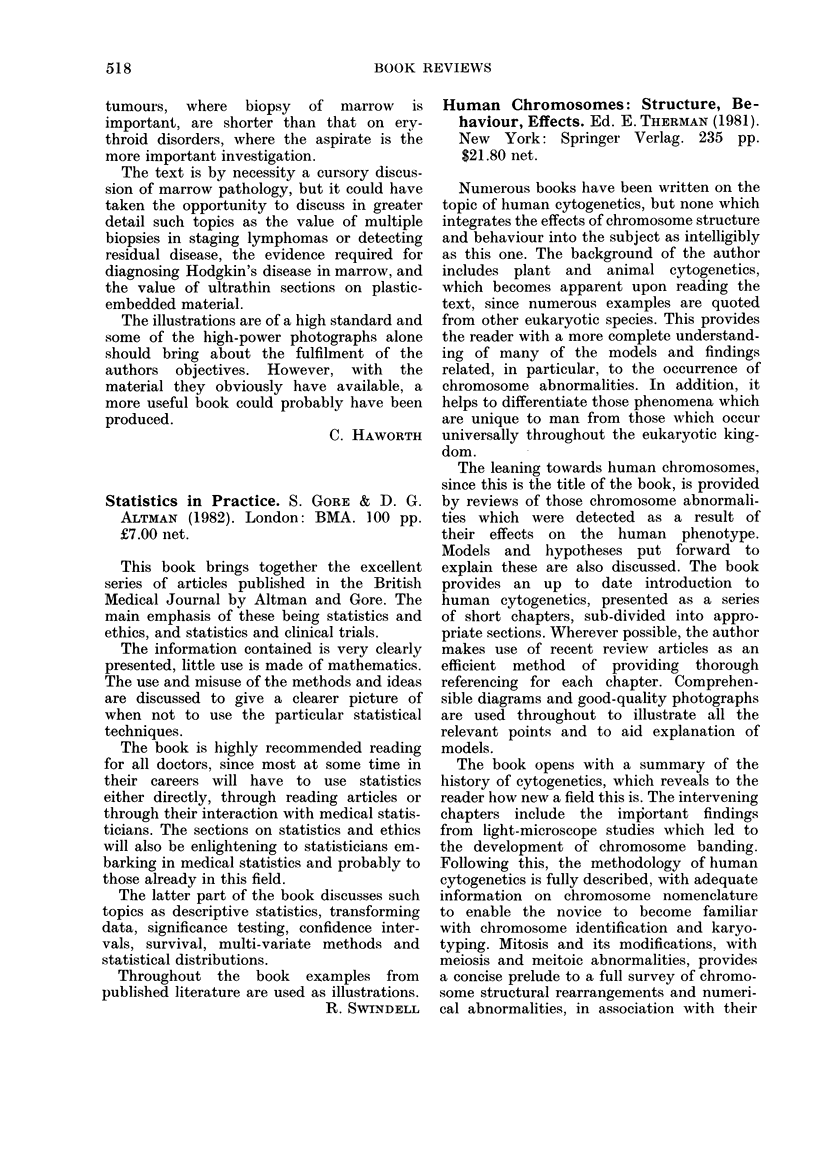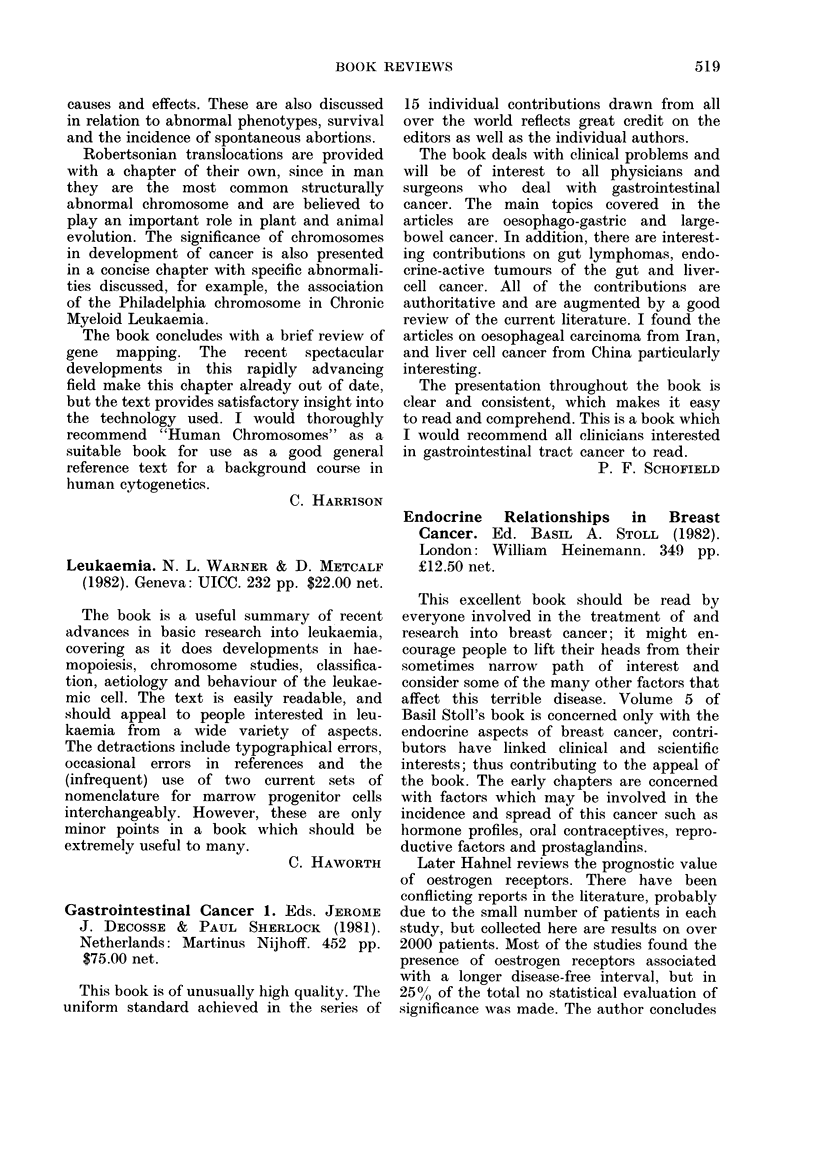# Human Chromosomes: Structure, Behaviour, Effects

**Published:** 1982-09

**Authors:** C. Harrison


**Human Chromosomes: Structure, Behaviour, Effects.** Ed. E. THERMAN (1981). New York: Springer Verlag. 235 pp. $21.80 net.

Numerous books have been written on the topic of human cytogenetics, but none which integrates the effects of chromosome structure and behaviour into the subject as intelligibly as this one. The background of the author includes plant and animal cytogenetics, which becomes apparent upon reading the text, since numerous examples are quoted from other eukaryotic species. This provides the reader with a more complete understanding of many of the models and findings related, in particular, to the occurrence of chromosome abnormalities. In addition, it helps to differentiate those phenomena which are unique to man from those which occur universally throughout the eukaryotic kingdom.

The leaning towards human chromosomes, since this is the title of the book, is provided by reviews of those chromosome abnormalities which were detected as a result of their effects on the human phenotype. Models and hypotheses put forward to explain these are also discussed. The book provides an up to date introduction to human cytogenetics, presented as a series of short chapters, sub-divided into appropriate sections. Wherever possible, the author makes use of recent review articles as an efficient method of providing thorough referencing for each chapter. Comprehensible diagrams and good-quality photographs are used throughout to illustrate all the relevant points and to aid explanation of models.

The book opens with a summary of the history of cytogenetics, which reveals to the reader how new a field this is. The intervening chapters include the important findings from light-microscope studies which led to the development of chromosome banding. Following this, the methodology of human cytogenetics is fully described, with adequate information on chromosome nomenclature to enable the novice to become familiar with chromosome identification and karyotyping. Mitosis and its modifications, with meiosis and meitoic abnormalities, provides a concise prelude to a full survey of chromosome structural rearrangements and numerical abnormalities, in association with their causes and effects. These are also discussed in relation to abnormal phenotypes, survival and the incidence of spontaneous abortions.

Robertsonian translocations are provided with a chapter of their own, since in man they are the most common structurally abnormal chromosome and are believed to play an important role in plant and animal evolution. The significance of chromosomes in development of cancer is also presented in a concise chapter with specific abnormalities discussed, for example, the association of the Philadelphia chromosome in Chronic Myeloid Leukaemia.

The book concludes with a brief review of gene mapping. The recent spectacular developments in this rapidly advancing field make this chapter already out of date, but the text provides satisfactory insight into the technology used. I would thoroughly recommend "Human Chromosomes" as a suitable book for use as a good general reference text for a background course in human cytogenetics.

C. HARRISON